# The moderating role of optimism between social trauma and depression among Chinese college students: a cross-sectional study

**DOI:** 10.1186/s40359-023-01314-z

**Published:** 2023-09-11

**Authors:** Jie Luo, Wei Cao, Jianhua Zhao, Xiaojin Zeng, Yun Pan

**Affiliations:** 1 School of Psychology, Guizhou Normal University, Guiyang, China; 2 School of Physical Education, Guizhou Normal University, Guiyang, China; 3 Guizhou Provincial Educational Science Academy, Guiyang, China; 4 Journal Editorial Department, Guizhou Normal University, Guiyang, China; 5 Guizhou Sports Vocational College, Guiyang, China

**Keywords:** Social trauma, Optimism, Depression, Moderation effect, Chinese college students

## Abstract

**Background:**

Although there is a robust relationship among social trauma, optimism, and depression, the inner mechanism of this correlation remains unclear and need to be further explored. The mainly purpose of the current study was to investigate the relationship between social trauma, optimism, and depression among college students in China. More specifically, examined the moderating role of the optimism between social trauma and depression in Chinese college students.

**Methods:**

A sample of 464 Chinese college students (54.7% female, M_age_=19.29) from three universities were selected by the convenient sampling, and the Social Trauma Questionnaire (STQ), the Optimism Questionnaire (OPQ), and the Self-Rating Depression (SDS) were completed by these Chinese undergraduates. The descriptive statistics, Pearson correlations, and hierarchical regression analysis were used to examine the results.

**Results:**

(1) The social trauma was positively associated with depression, whereas the optimism was negatively associated with social trauma, and depression; (2) The social trauma had a significant correlation with depression, and the optimism could moderate the relationship between social trauma and depression. More specifically, the further study showed that there was a significant positive relation between social trauma and depression under the low optimism level, however, there was a non-significant relation between social trauma and depression under the high optimism level.

**Conclusion:**

The optimism is the protective mechanism of college students’ mental health (e.g., depression), it could weaken the trauma that associated with social trauma among college students.

## Introduction

The emerging adulthood [1], a term initially proposed by Arnett (2000), is a new theory of development from the late teens through the twenties, and is the distinct period between adolescence and young adulthood, with a focus on ages 18–25 [1]. According to Arneet, the emerging adulthood distinguished by relative independence from social roles and from normative expectations. Having left the dependency of childhood and adolescence, and having not yet entered the enduring responsibilities that are normative in adulthood, emerging adults often explore a variety of possible life directions in love, work, and worldviews [1]. In addition, some research suggests that the emerging adulthood is an emotionally complex period during which people develop careers and niches in society, enter adult relationships, and encounter potentially high levels of stress because of family, financial, and career obligations [2, 3], as well as often experience emotional turmoil [4]. It is, therefore, important to help emerging adults express more positive emotions and vent negative affect properly to increase their ability to deal with life’s pressure and challenges.

Indeed, the university stage happens to be in this period (i.e., emerging adulthood). The college students are in the transition from teenagers to adults, facing various kinds of pressure and challenges (e.g., interpersonal relationship, competitive pressure, academic burden, and future planning) which result in the high incidence of depression, a potent factor affecting mental health [5–8]. A meta-analysis demonstrated that the detection rate of depressive symptoms of Chinese college students is 24.71% [8]. According to the recent findings of 2022 blue book on national depression, the incidence of depressive symptoms is trending younger, with 35.32% of Chinese people aged 18–24 years old suffering from depression symptoms [9]. The clinical manifestations of depression including slow mentality, low mood, insomnia, and slow movement [10]. What’s more, this phenomenon appears related to increased learning stress, decreased interest in learning, declined subjective well-being and impaired social function, which would turn out to be severe barrier in college students’ daily life [10–13]. Considering that the depression would have a negative impact on cognition, behavior, and social function of college students, if not timely intervention and counseling, it would increase the degree of depression, and even evolve into clinical depression (i.e., depressive disorder), which would cause risky behaviors (e.g., self-injury and suicide) [13–16]. Hence, the prevention and treatment of depression has become an important work in the mental health education of college students, and how to effectively restrain the depression of college students has become a key content of mental health education in colleges and universities under the strategic background of healthy China.

### Social trauma and depression

According to the social ecosystem theory, individual psychological and behavioral development is the result of continuous interaction between the social environment system and the individual internal system [17]. The social environment is an important external factor affecting the emotion and behavior of people, which interacts with individual factors and jointly restricts the level of mental health. Social trauma is defined as a series of emotionally directed and stressful events that individuals personally perceived as stressful and even potentially overwhelming, because these events occur repeatedly, continuously, or accumulatively during social interactions and social competition, such as poor interpersonal relationships, being bullied, being neglected, and being threatened [18]. According to Zhang et al. (2017), the social trauma includes three sub-types (i.e., social exclusion, over-control, and weakness in social competition). More specifically, the first sub-type is “social exclusion or alienation” which means individuals are not valued or respected in real social situations, they perceive much more rejection, discrimination, exclusion, unfair treatment, disregard, suspicion, deception, or deliberate abandonment. The second sub-type is “being overly control” which refers the relationships of individuals with friends, spouses, peers, colleagues, or supervisors is a kind of unequal relationship. The third sub-type is “weakness in social competition” which represents powerlessness, disadvantage, or lack of voices during social competition for opportunities of learning, job, and status, all of which refer to social resources. Additionally, unlike the first two sub-types, the third subtype is not directly attribute to other people’ negative responses but is caused by the blockage of “unidirectional drive upward” during social comparison [18]. Prior investigation has showed that the scores of social trauma of Chinese vocational college students were higher than undergraduates, soldiers, and adults from the community. Moreover, social trauma was significantly correlated with various mental health-related indexes, such as sense of control, insecurity, happiness, anxiety and depression symptoms, and PTSD severity [18].

Indeed, aforementioned adversities would cause psychological trauma and give rise to numerous negative outcomes [17–20], activation of dysfunctional attitudes, cognitive bias, distortion, and ultimately lead to depression [18, 19]. According to the Cumulative Risk Model (CRM), a variety of risk factors encountered by individuals in the process of growth will damage their cognition system or emotion system, even behavior system. All mental systems of individuals isn’t sensitive to a single risk factor, however, when the risk factors accumulated, it can lead to anxiety, depression or deviant behaviors, that is, the accumulated risk factors that individuals encounter in the daily life is the direct causes of physical and mental health problems [20–24]. Social adversity is a negative cumulative factor in the individual’s growth process. When these adverse events are accumulated to a certain extent, it will cause various kinds of inappropriate developmental problems for individuals, such as depression, aggressive behavior, and criminal behavior [25]. Other research implies that accumulative social adversities could not only result in the destruction of the value system, but also the decreases of self-esteem, sense of insecurity and other individual PTSD symptoms simultaneously [18, 26]. Likewise, social trauma was significantly correlated with various mental health related indices, such as sense of control, insecurity, anxiety, and depression symptoms [18]. In addition, the interpersonal theory of depression suggests that individuals in bad interpersonal relationships lack of a sense of belonging due to their interpersonal needs cannot be met, which induces depression [27]. previous studies have indicated that the negative life events experienced by college students have a positive predictive effect on depression [28, 29]. In summary, from the standpoints of prior literatures, we proposed the Hypothesis 1: Social trauma could significantly correlate with the depression of college students.

### The moderation effect of optimism

**Fig. 1:**
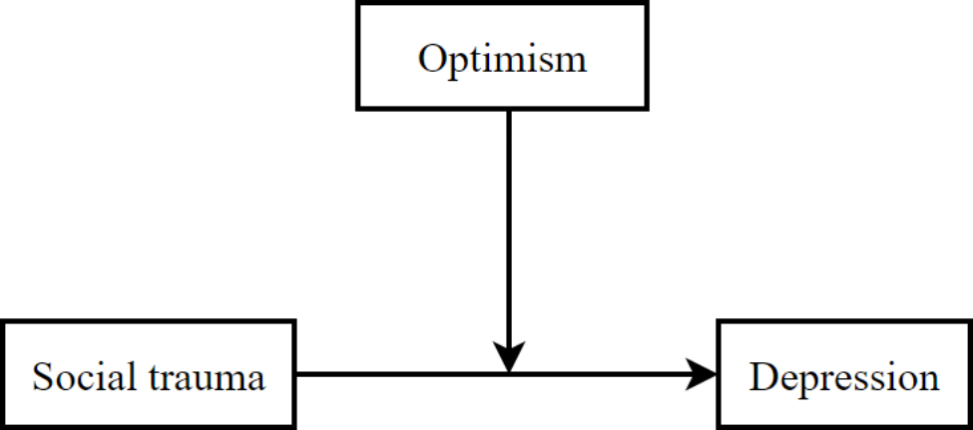
Hypothesized research model

As a positive personal trait, the optimism refers to an inclination to put the most favorable construction upon actions and events or to anticipate the best possible outcome [30, 31]. Pervious research indicates that the optimism might serve as a protective factor to maintain individual’s mental health, and obviously could buffer against the effects of negative stimulus to human beings [31–34]. In other words, the individuals who are considered to be optimists would look forward to a positive outcome, and thus experience less depression and anxiety, whereas the individuals who are considered to be pessimists would tend to focus on the adverse aspect of life and preferentially use more pessimistic interpretations, thus experience more negative emotions and hard to succeed [34–38]. As relevant researches demonstrated that the optimism played a moderating role in the association between online rejection and depression, rumination and suicidal ideation, upward social comparison and depression among college students [38–41]. Accordingly, the current study speculated that the optimism could significantly moderate the relationship between the social trauma and depression. In other words, the optimism could mitigate or decrease the association. Therefore, we proposed the Hypothesis 2: Optimism could significantly negative associate with depression, and it would moderate the relationship between the social trauma and depression among college students (see Fig. 1).

### The current study

To sum up, we proposed that there is a robust relationship among social trauma, optimism, and depression among college students. Nevertheless, the inner mechanism of this correlation remains unclear and need to be further explored. Therefore, a particular focus of the present study was to clarify the potential role of the optimism in the relationship between the social trauma and depression among college students. More specifically, this study was to investigate the effect of social adversity on depression, and the moderating role of optimism between this relationship, to further explain and clarify the related risk and protective factors of depression among college students. In order to ultimately help college students shape their optimistic quality and promote their healthy growth and development.

## Methods

### Participants and procedure

We used convenient sampling method to recruit participants from three universities in Guizhou Province of China. In the present study, we distributed 500 questionnaires, and there were 36 invalid questionnaires that were deleted. The valid sample without missing data included 464 questionnaires, of which were 210 males (45.3%) and 254 females (54.7%). There were 345 freshmen (74.4%), 64 sophomores (13.8%), 31 juniors (6.7%), and 24 seniors (5.2%). The participants’ age ranged from 18 to 25 years with a mean age of 19.29 (*SD* = 1.33).

The study was approved by the Institutional Review Board of the Guizhou Normal University. All the participants provided written consent prior to completing the questionnaire. The participants were informed that the study was voluntary, and they could discontinue at any time. In addition, the participants were informed that their answers would remain anonymous and were invited to ask questions regarding the investigation. Finally, the participants completed the questionnaires with pencils or pens during the class under the supervision of teachers and researchers. After completion, all participants would get a gift showing our gratitude.

### Measures

#### Social Trauma Questionnaire (STQ)

The Social trauma was measured by the Social Trauma Questionnaire (STQ) [18], which was aimed to assess one’ s perception of different aversive situations in daily social life. The STQ consists of 28 items, and is divided into 3 dimensions: social exclusion, overcontrol, weakness in social competition. The STQ is rated on 5-point Likert scale (1 = strongly disagree, 5 = completely agree). In the current study, the overall Cronbach’s α coefficient of the scale is 0.957 and the reliability coefficients of the three subscales ranged from 0.859 to 0.933. In addition, the CFA suggested that the three factors model of the STQ has adequate construct validity (χ^2^*/df* = 4.716, CFI = 0.958, TLI = 0.954, RMSEA = 0.089).

#### Optimism Questionnaire (OPQ)

The optimism was measured by Optimism Questionnaire (OPQ) [42], which was aimed to assess individual level of optimism. The OPQ consists of 16 items, and is divided into 3 dimensions: positive coping, positive cognition, and negative trend. The OPQ is rated on 5-point Likert scale (1 = strongly disagree, 5 = completely agree). In the current study, the overall Cronbach’s α coefficient of the OPQ is 0.898 and the reliability coefficients of the three subscales range from 0.812 to 0.935. Additionally, the CFA indicated that the three factors structure of the OPQ has adequate construct validity (χ^2^*/df* = 3.244, CFI = 0.963, TLI = 0.955, RMSEA = 0.052).

#### Self-Rating Depression Scale (SDS)

The depression was measured by Revised Self-Rating Depression with Chinese Edition (SDS) [43, 44], which was designed to assess one’s depression in daily life. The SDS consists of 20 items, and is rated on 4-point Likert scale (1 = never, 4 = always). In the current study, the overall Cronbach’s α coefficient of the SDS is 0.827. In addition, the CFA showed that the SDS has acceptable construct validity (*χ*^*2*^*/df* = 5.074, CFI = 0.921, TLI = 0.910, RMSEA = 0.094).

### Statistical analysis

Firstly, we used SPSS 25.0 to test the common method bias (CMB) with Harman’s single-factor method [45]. Secondly, we calculated descriptive statistics (e.g., *M*, *SD, SK, and KU*) of key variables (i.e., social trauma, optimism, and depression), Pearson correlations, reliability coefficients, and the construct validity of the three questionnaires by means of confirmatory factor analysis with Mplus 8.0 and so one. Thirdly, we used hierarchical regression analysis (HRA) [46] and the SPSS PROCESS macro, Model 1 (Version 3.0) [47] to test the moderation of the optimism between social trauma and depression. Finally, we used plot regression lines to calculate different level of optimism and its relation between social trauma and depression: the mean of the variable and points one standard deviation above and below the mean.

## Results

### Common method variance analysis

We tested common method variance with Harman’s single-factor method. The results showed that there were 11 common factors being extracted, and the first common factor explained 27.95% of the total variance, and it was less than 40% [45]. Thus, the current study didn’t exist serious common method variance problems.

### Descriptive statistics and Pearson correlation

The descriptive statistics of the key variables, and Pearson correlations were conducted to examine the relationships among social trauma, optimism, and depression (see Table 1). The results showed that social trauma was positively correlated with depression (*r* = 0.429, *p* < 0.001), and social trauma was negatively correlated with optimism (*r*=-0.492, *p* < 0.001); additionally, depression was negatively correlated with optimism (*r*=-0.634, *p* < 0.001).

**Table 1:** The descriptive statistics and Pearson correlations of the study variables

Variables	*M*	*SD*	*SK*	*KU*	ST	OP	Dep
ST	50.24	15.00	0.407	-0.114	0.957		
OP	61.80	9.73	-0.223	-0.328	-0.492^***^	0.898	
Dep	37.45	7.84	0.090	-0.911	0.429^***^	-0.634^***^	0.827

M, mean; SD, standard deviation; SK, skewness; KU, kurtosis; ST, social trauma; OP, optimism; Dep, depression;^***^p < 0.001; The values on the diagonal are the coefficient αs of each questionnaire

### Moderating effect analyses

Following the moderating effect test procedure [46], the hierarchical regression analysis was used to test the moderation of optimism with SPSS25.0. More specifically, to avoid multicollinearity, all the variables were standardized. After standardizing the independent variable (ST), the moderating variable (OP), and the dependent variable (SDS), the product term (ST × OP) of the standardized independent variable and the moderating variable is taken as the independent variable for hierarchical regression analysis (see Table 2).

**Table 2:** The moderation of optimism between social struma and depression

							Depression					
	Variables		*R* ^2^	ρ*R*^2^		ρ*F*		*p*	B (SE)	β	*t*	*p*
Step 1	ST		0.420	0.420		166.835^***^		< 0.001	0.159 (0.042)	0.155	3.808^***^	< 0.001
	OP								-0.560 (0.041)	-0.557	-13.683^***^	< 0.001
Step 2	ST		0.426	0.006		4.496^*^		0.035	0.155 (0.042)	0.151	3.720^***^	< 0.001
	OP								-0.551 (0.041)	-0.549	-13.445^***^	< 0.001
	ST×OP								-0.079 (0.037)	-0.076	-2.120^*^	0.035

ST, social trauma; OP, optimism; ρR^2^, R square change; ρF, F change, B, Unstandardized coefficients; SE, Standard error; β, Standardized coefficients;^*^p < 0.05;^***^p < 0.001

The results showed that: (1) Social trauma could positively predict depression (β = 0.155, *p* < 0.001); (2) Optimism could predict depression negatively (β=-0.557, *p* < 0.001); (3) Interaction (social trauma × optimism) could also negatively predict depression (β=-0.076, *p* = 0.035). Furthermore, the moderation model was implemented through the SPSS PROCESS macro with Model 1 [47], and the moderating effect was determined on the basis of 5,000 bootstrap samples in generating 95% bias-corrected bootstrap confidence intervals [47]. The results indicated that the social trauma had a significant positive prediction on depression (B = 0.155, SE = 0.042, 95% CIs: 0.073 ~ 0.236), while the optimism had significant negative prediction on depression (B=-0.551, SE = 0.041, 95% CIs: -0.632~ -0.471); and the interaction (social trauma × optimism) also had a significant negative prediction on depression (B=-0.079, SE = 0.037, 95% CIs: -0.151~ -0.006). Overall, these results suggested that the optimism might moderate the relationship between social trauma and depression among Chinese college students.

**Fig. 2:**
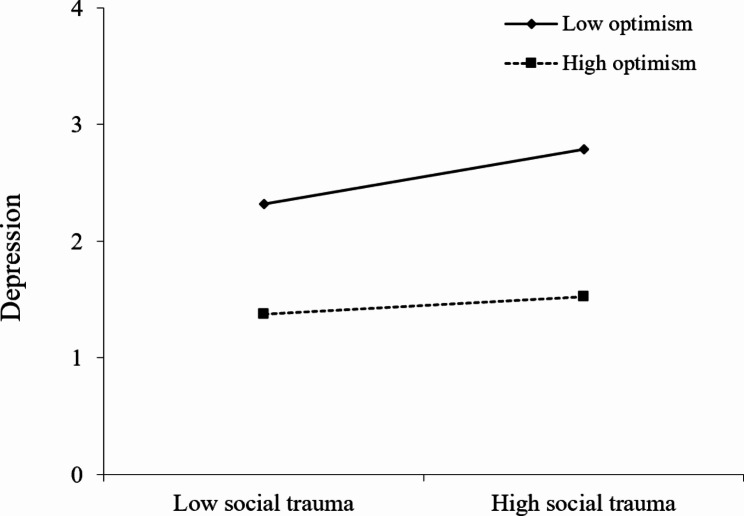
The plot of simple slopes of optimism

Then, to examine how the optimism moderates the relationship between social trauma and depression, the simple slopes were plotted for the values of high optimism (1 SD above the mean) and low optimism (1 SD below the mean), as shown in Fig. 2. The results indicated that, for the group with low level of optimism, there was a significant positive relationship between social trauma and depression (Β = 0.360, SE = 0.063, *t* = 5.763, *p* < 0.001, 95% CIs: 0.236 ~ 0.485), while this relationship was not significant for group with high level of optimism (Β=-0.001, SE = 0.114, *t*=-0.006, *p* = 0.996, 95% CIs: -0.228 ~ 0.226).

## Discussion

### Social trauma and its relation to depression

Consistent with previous researches [18, 24, 25], social trauma was significantly associated with depression among college students which supports for Hypothesis 1. The social trauma refers to a series of emotionally directed and stressful events that people personally perceived as stressful and even potentially overwhelming because these events occur repeatedly, continuously, or accumulatively during social interactions and social competition [18]. Obviously, the higher the degree of social trauma suffered by college students, the more prone to depression, and social trauma can significantly positively predict the depression of college students. The information of adversity perceived by college students would cause a kind of non-life safety psychological trauma, thus affecting their mental health level (i.e., depression) [20]. When college students encounter social adversity, they would experience negative emotions such as anxiety, depression, and tension, which would also cause chronic trauma including reduced self-esteem, wavering value belief and increased stress level [18, 26].

In addition, according to the hopelessness theory [48], depression not only is related to the perception level of the social chronic adversity but also the cognitive evaluation processing of individuals. College students with high social trauma are usually towards pessimistic cognitive evaluation processing [48], they might be more likely to attribute their failure to internal aspect, and thus deviate from the target. By leading to a series of negative emotions, this frustration could bring about repeatedly thinking about the reason for and consequences of the distress, excessive attention on the adversities and finally defect individual’s cognitive control [49]. By leading to rumination, they usually catastrophize the aversive information. In return, it will exacerbate the extent of social trauma. This vicious circle brings about learned helplessness, which refers to a set of motivational, cognitive, emotional, and behavioral deficits including low achievement motivation, low self-efficacy, negative thinking, no willpower, hopeless and impaired social function. Without promptly psychological intervention and treatment, the consequences are of special serious. However, College students with low social trauma tend to optimistic evaluation processing, they would not abandon or deny themselves causally when they encounter social adversities, and try their best to solve the difficulties they meet. At the same time, they have positive personality traits and optimistic attitude towards social life. Finally, the general stress theory suggested that after experiencing social traumatic events, individuals might suffer huge psychological pressure, which makes individuals produce one even more negative emotion. Overall, social trauma, as a risk factor, has a direct predictive effect on depression in college students.

### The moderation effect of Optimism

Statistics implicated, serving as a correlation with depression among college students, optimism also moderated the correlation between social trauma and depression which supports for Hypothesis 2. College students with lower levels of optimism, the correlation between social trauma and depression was higher. Whereas for those with higher levels of optimism, the relationship was non-significant, which is consistent with the prior researches [38–41, 50]. As a positive psychological quality, the optimism refers to individuals have a positive attitude and expectation towards the future events, and a positive attitude towards coping with difficulties in life [31]. Under the stressful situations and social adversity, college students with higher level of optimism tend to take positive coping strategies to relieve stress and to maintain physical and mental health [35]; when facing difficulties and social stressful events, people with higher level of optimism are full of confidence in the future and themselves, they dare to face the problems, strive to find solutions to the problems, and can persevere and go all out [33–36]. Moreover, combined hopelessness theory [48] and buffering model theory [50–53], we can further explore how optimism works on the relations between social trauma and depression. When facing more social adversities in their social life, the college students will repeatedly consider about the causes and consequences of the situation. As time goes by, those accumulated negative social adversities will activate the previous negative memory and then result in helplessness and depression. However, optimism operates as a protective buffer in this process which can attenuate the reaction caused by negative stimulus and then protect individual’s mental health. For college students with high level of optimism, the buffering effect of optimism is even stronger. When they encounter with social adversities, the optimistic buffering mechanism will enable them to solve the difficulty with positive attitudes and coping strategies, that is, optimism is a kind of catalyst with positive energy to enable those individuals to be stronger and tougher than ever when they encounter with social adversities, and thus they are far less likely to depress, while for college students with low level of optimism, optimism is a kind of catalyst with negative energy, it can result in more desperate and more pessimistic or even more depressive than before when they face with social adversities, and thus experience more negative emotions and showing a strong tendency of depression [35, 37]. Overall, optimism, as a protective factor, it could buffer the effect of social trauma on depression in college students.

### Implication

The findings of the present study have some important implications for both theoretical and practical research settings. First, our results support that college students’ mental health (e.g., depression) is the result of continuous interaction between social environment system (e.g., social trauma) and individual internal system (e.g., optimism). Furthermore, to decrease the incidence of depression among college students, we could consider in two aspects. On the one hand, universities need to purify the learning and living environment, so as to reduce the frequency of adverse events and social trauma of the college students. Mental health in colleges and universities should consciously focus on the college students with social traumatic experiences, and provide psychological counseling to them in time, so as to alleviate the psychological harm brought by social traumatic events to college students and prevent from depression in advance. On the other hand, both universities and families should strengthen the cultivation of students’ optimism, so then they will be ready to encounter with the failure and frustration easily and experience less negative emotion.

### Limitations and future direction

The research must be considered within the context of the limitations below. Firstly, this study participants were recruited predominantly from Guizhou Province, Southwest China; and the demographic variables were somewhat unbalanced (in grades, specifically); future work should attempt to replicate and expand the findings of this study to other Chinese regions, and try to keep the balanced sample sizes across grades when recruiting participants. Secondly, this research was cross-sectional in design, which implicates it failed to demonstrate the dynamic relationship of social trauma, optimism, and depression of college students. Theoretically speaking, testing via longitudinal research would be more suitable. Finally, besides of optimism, whether there is other factor could mediate or moderate the relationship between social trauma and depression need to be further studied and examined.

## Conclusion

Despite these limitations, results of the current study have suggested that the social trauma was positively associated with depression, whereas the optimism was negatively associated with social trauma, and depression; moreover, the optimism could moderate the relationship between social trauma and depression. More specifically, the optimism is the protective mechanism of college students’ depression, it could weaken the trauma that associated with social trauma among college students.

## Data Availability

The data supporting the conclusions of this study are available upon request to the first author, Jie Luo.
